# Increased Presentation of Diabetic Ketoacidosis and Changes in Age and Month of Type 1 Diabetes at Onset during the COVID-19 Pandemic in Spain

**DOI:** 10.3390/jcm11154338

**Published:** 2022-07-26

**Authors:** Isabel Leiva-Gea, Cristina Antúnez Fernández, Roque Cardona-Hernandez, Marta Ferrer Lozano, Pilar Bahíllo-Curieses, Javier Arroyo-Díez, María Clemente León, Maria Martín-Frías, Santiago Conde Barreiro, Andrés Mingorance Delgado, Jacobo Pérez Sánchez

**Affiliations:** 1 Pediatric Endocrinology, Department of Pediatrics, Hospital Regional de Málaga, 29010 Málaga, Spain; 2 Instituto de Investigación Biomédica de Málaga (IBIMA), 29590 Málaga, Spain; 3 Department of Pediatrics, Hospital Punta de Europa, 11207 Algeciras, Spain; 4 Division of Pediatric Endocrinology, Hospital Sant Joan de Déu, Passeig Sant Joan de Déu 2, 08950 Barcelona, Spain; 5 Pediatric Endocrinology, Department of Pediatrics, Hospital Universitario Miguel Servet, 50009 Zaragoza, Spain; 6 Pediatric Endocrinology, Department of Pediatrics, Hospital Clínico Universitario de Valladolid, 47003 Valladolid, Spain; 7 Pediatric Endocrinology, Department of Pediatrics, Hospital Universitario Materno Infantil de Badajoz, 06010 Badajoz, Spain; 8 Pediatric Endocrinology, Department of Pediatrics, Institut de Recerca, Hospital Vall d’Hebron, Centre for Biomedical Research on Rare Diseases (CIBERER), 08035 Barcelona, Spain; 9 Barcelona Autonomous University, 08035 Barcelona, Spain; 10 Pediatric Endocrinology and Diabetes, Department of Pediatrics, Hospital Universitario Ramón y Cajal, 28034 Madrid, Spain; 11 Pediatrics, Centro de Salud de Barbastro, 22300 Huesca, Spain; 12 Pediatric Endocrinology, Department of Pediatrics, Hospital General Universitario de Alicante, 03010 Alicante, Spain; 13 Pediatric Endocrinology, Department of Pediatrics, Consorci Coprporació Sanitaria Parc Tauli, 08208 Sabadell, Spain

**Keywords:** type 1 diabetes, COVID-19, diabetes onset, DKA

## Abstract

**Objective**: To assess the impact of the COVID-19 pandemic and lockdown measures on the presenting characteristics (age at diagnosis, severity, monthly distribution) of newly diagnosed type 1 diabetes in Spanish children. **Research Design and Methods**: An ambispective observational multicenter study was conducted in nine Spanish tertiary-level hospitals between January 2015 and March 2021. Inclusion criteria: new cases of type 1 diabetes in children (0–14 years) recording age, sex, date of diagnosis, presence of diabetic ketoacidosis (DKA) at onset, and severity of DKA. Data were compared before and during the pandemic. **Results**: We registered 1444 new cases of type 1 diabetes in children: 1085 in the pre-pandemic period (2015–2019) and 359 during the pandemic (2020–March 2021). There was a significant increase in the group aged ≤4 years in the pandemic period (chi-squared = 10.986, df 2, *p* = 0.0041). In 2020–2021, cases of DKA increased significantly by 12% (95% CI: 7.2–20.4%), with a higher percentage of moderate and severe DKA, although this increase was not significant. In 2020, there was a sharp decrease in the number of cases in March, with a progressive increase from May through November, higher than in the same months of the period 2015–2019, highlighting the increase in the number of cases in June, September, and November. The first three months of 2021 showed a different trend to that observed both in the years 2015–2019 and in 2020, with a marked increase in the number of cases. **Conclusions**: A change in monthly distribution was described, with an increase in DKA at onset of type 1 diabetes. No differences were found in severity, although there were differences in the age distribution, with an increase in the number of cases in children under 4 years of age.

## 1. Introduction

Epidemiological studies have an important role in type 1 diabetes, since they enable estimation of the necessary resources for its management, as well as providing insight into its etiology and risk factors.

The estimated incidence of type 1 diabetes in the Spanish population under 15 years of age is 17.69 cases/100,000 inhabitants/year [1]. In Spain, incidence figures are lower in autonomous communities located in the north of the country, and higher in the south and center of the country, suggesting that the “north–south” gradient in the incidence of the disease described in Europe does not apply [2].

On 31 January 2020, the first case of coronavirus disease 2019 (COVID-19) was diagnosed in Spain [3]. From this time onwards, the incidence of the virus skyrocketed, leading to the declaration of a state of alarm and home lockdown on 14 March 2020. An association between COVID-19 and an increase in the number of hyperglycemia or diabetes cases has been suggested based on findings from different observational studies around the world. Numerous hypotheses have been proposed, although the mechanism that links them is not clearly defined [4]. Angiotensin-converting enzyme receptor 2 (ACE2) is the binding site for SARS-CoV-1 and -2 and is strongly expressed in pancreatic endocrine cells. Therefore, some studies postulate that the exposure to SARS-CoV-2 contributes to the observed increase of cases by precipitating or accelerating the onset of type 1 diabetes [5,6]. Another suggested mechanism would be the direct action of COVID-19 on pancreatic beta cells, by increasing proinflammatory cytokines and acute phase reactants leading to inflammation and direct cell damage.

A number of studies examining the presentation of new cases of type 1 diabetes in children and adolescents after the declaration of the pandemic, reported an increase in the frequency severity of diabetes ketoacidosis (DKA) presentation [6,7,8,9,10,11,12]. On the other hand, some studies have described an increase in the frequency of new case presentations [6,10,11,12,13,14,15,16], while others reported no increase or even a decrease in the number of type 1 diabetes [9,17].

The change in the frequency and severity in type 1 diabetes forms presentation after the emergence of the pandemic, raises the hypothesis that variations in the circulation of seasonal viruses, which could have been acting as triggers for onset or the delay in diagnosis due to individuals postponing consultation because of the fear of infection, may have contributed to this new scenario [9].

The aim of the present study was to assess the impact of the COVID-19 pandemic and the resulting lockdown measures on the presenting characteristics of newly diagnosed type 1 diabetes in Spanish children with respect to age at diagnosis, severity, and monthly distribution.

## 2. Research Design and Methods

This was a multicenter, observational, ambispective study conducted in nine Spanish tertiary-level hospitals between January 2015 and February 2021. We included all new diagnoses of type 1 diabetes in the pediatric population (0–14 years) in each of the participating centers. Age, sex, date of diagnosis, presence of DKA at onset, and severity of DKA (blood pH, serum bicarbonate) were recorded. Data were collected in two periods and compared before the pandemic (2015–2019) and during the pandemic (2020–2021).

The study was undertaken with the approval of the Ethics Committee of the Regional Hospital of Malaga. The diagnostic criteria for type 1 diabetes were those established by the American Diabetes Association [18]. The criteria used to define the severity of DKA followed the recommendations of the International Society for Pediatric and Adolescent Diabetes (ISPAD) [19] as follows: mild if pH < 7.3 or bicarbonate < 15 mmol/L, moderate if pH < 7.2 or bicarbonate < 10 mmol/L, and severe DKA if pH < 7.1 or bicarbonate < 5 mmol/L.

The statistical analysis was performed using R Commander Version 2.7-1 software (Chapman & Hall, Boca Raton, FL, USA). To evaluate the differences between the different study years, the chi-squared test was used, with *p* < 0.05 being considered statistically significant. The resulting data are presented in tables and figures to illustrate the distribution of the data.

## 3. Results

We collected data from 1444 new cases of type 1 diabetes in the pediatric age group during the period 2015–2021: 1085 in the pre-pandemic period (2015–2019) and 359 during the pandemic (2020–March 2021). Regarding the distribution of cases by age, an increase in the group aged ≤4 years was evident in the pandemic period, with a chi-squared value of 10.986 with 2 degrees of freedom and a *p*-value of 0.0041 (Table 1), indicating that the age distribution of new cases was not the same in these periods. This significant difference was at the expense of more cases (25%) in children under 4 years of age during the pandemic rather than in the pre-pandemic period (19%). The opposite occurred in the group aged 5–9 years, where there was a higher percentage in the first period (39%) rather than in the second (30%). During the period 2015–2019, 589 boys (54%) and 494 girls (46%) presented with type 1 diabetes; meanwhile, during 2020–2021, 205 boys (57%) and 154 girls (43%) did. No differences in gender distribution were found according to the period when diabetes onset occurred (X-square 0.754, df 0.38).

**Figure 1:**
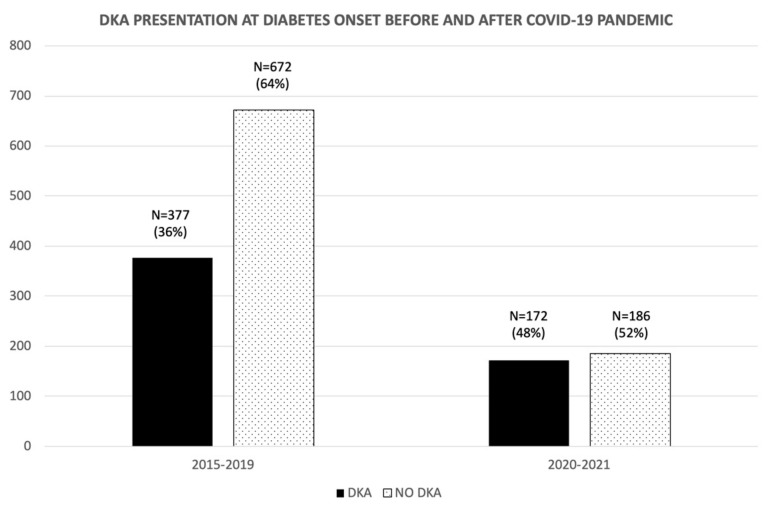
Percentage of DKA as a form of presentation during time periods 2015–2019 versus 2020–2021. Episodes are expressed in number and percentage (%).

**Figure 2:**
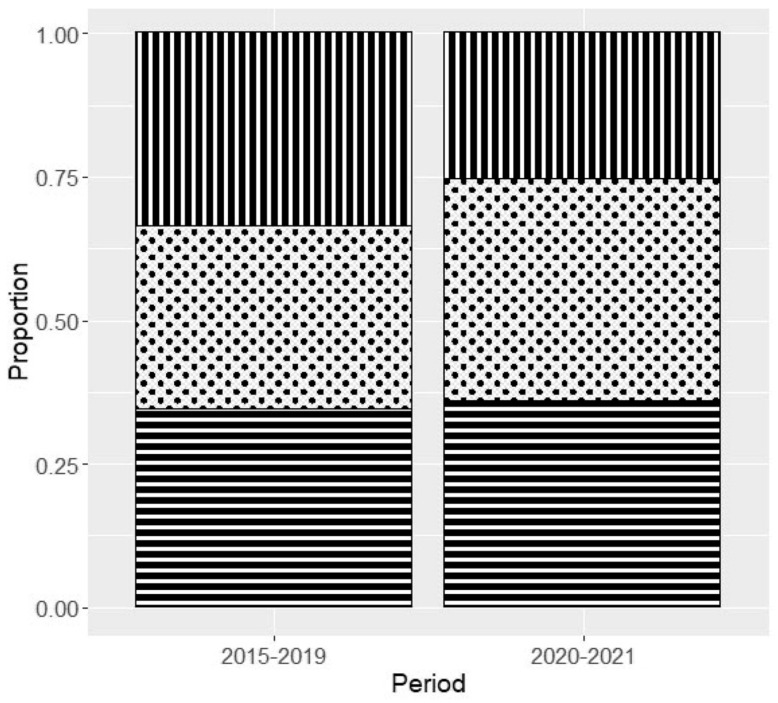
Proportion of cases by DKA severity during periods 2015–2019 and 2020–2021. Each square represents DKA severity cases. Squares filled with vertical black bars represent mild cases; Squares with black dots represent moderate cases. Squares filled with horizontal black bars represent severe cases.

During 2020–2021, the number of DKA cases increased significantly by 12% (95% CI: 7.2 to 20.4%) (Figure 1 and Figure 2). In this period, 48% of new-onset cases presented DKA (26% mild, 38% moderate, and 36% severe), whereas during the 2015–2019 period, a lower percentage of DKA was reported, 36% (33% mild, 32% moderate, and 34% severe). A higher percentage of moderate and severe DKA cases were seen in this period, although this increase was not significant (Figure 2).

**Figure 3:**
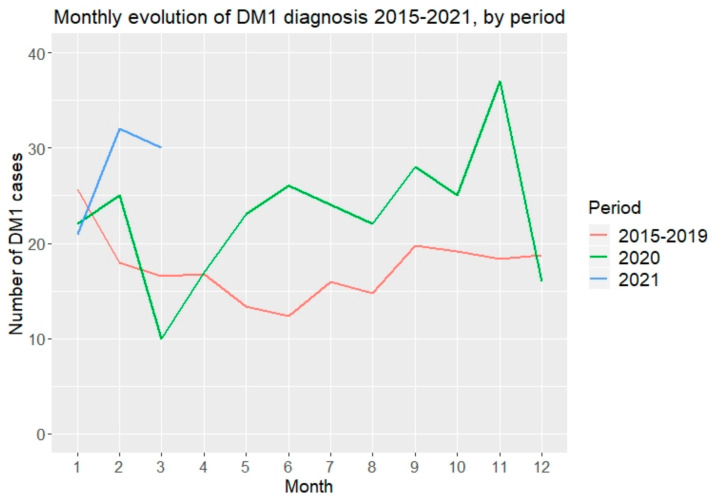
Monthly distribution of newly diagnosed type 1 diabetes cases by periods. Black line represents the cases by month for the period 2015–2019. Dotted line represents the cases by month during the year 2020. Dashed line represents the cases during the months of January, February, and March 2021.

The monthly distribution of new type 1 diabetes cases, as well as cases with DKA as a form of presentation, are shown in Figure 3. From 2015 to 2019, a dip in the number of cases from March through September was noted. In 2020, a sharp decrease in the number of cases diagnosed in March was observed, with an ulterior progressive increase from May through November, which was higher than in the same months of the period 2015–2019, with a notable increase in the number of cases during the months of June, September, and November. The first three months of 2021 showed a very different trend than the observed during the period 2015–2019 and in 2020, respectively, with a marked increase in the number of cases (Figure 3).

## 4. Discussion

The COVID-19 pandemic has had a major impact on our society, generating changes that may have influenced the epidemiological situation of other diseases such as the onset of type 1 diabetes. Our study describes an increase in cases of new-onset type 1 diabetes with DKA presentation in children and adolescents under 14 years of age in the period 2020–2021, following the declaration of the COVID-19 pandemic by the WHO, compared to previous years. No differences in severity were found over the entire 2020–2021 period. This differs from observations made by other authors from Germany, Italy, Australia, and Canada during the first months after the start of the pandemic [6,7,8,9,10,11,12,13] who described an increase in moderate and severe forms of DKA. The fact that the first descriptions in this regard refer to periods immediately after lockdown may have conditioned these findings due to delayed use of health care during the beginning of the pandemic and the initial restrictions. We can speculate that this finding may lay on the fact that many patients delay or avoid visits to hospitals due to fear of getting infected with SARS-CoV-2. We can also speculate with a higher awareness of diabetes onset symptoms among primary care pediatricians from Spain that was quickly reinforced by the announcement of the International Society for Pediatric and Adolescent Diabetes (ISPAD) statement on COVID-19 and the increased risk of developing severe forms of DKA at onset [20] may have influenced this fact.

We also found significant differences in the distribution by age group, with an increase in 2020–2021 of children diagnosed under 4 years of age. A study carried out in Germany showed a significant increase in both the incidence of type 1 diabetes and severe forms of DKA, with these results being more striking in children under 6 years of age [7]. The latter leads us to consider that SARS-CoV-2 may potentially be acting as a trigger for the autoimmune destruction which accelerates the process rather than inflicting direct damage to the beta cell, as younger age groups tend to have a more rapid and intense beta cell loss [21]. In addition, we can hypothesize that a modification in the presentation pattern of other seasonal infective agents, such as syncytial respiratory virus of flu due to the pandemic, may be altering the diabetes age at diagnosis.

A study conducted by the Italian Society of Pediatric Endocrinology and Diabetes assessing the epidemiological change during the Italian lockdown (20 February to 14 April 2020), in comparison with the same period in 2019, observed a decrease in the incidence of type 1 diabetes in the confinement period, with the overall incidence being similar in both years [9]. A subsequent analysis, limited to the Lombardy region [13], which included the presentation of new-onset cases during 2020, reported an increase in the number of cases with respect to 2017 and 2018 but not 2019. An evaluation of the monthly distribution in our data shows a different distribution in both 2020 and 2021, which does not correspond to the monthly distribution of recent years in Spain. In the second quarter of 2020, coinciding with the lockdown period in Spain, there was a striking decrease in the number of cases in the months of March and April. These results are aligned with those from Germany, Italy, and Finland [9,12,17]. In contrast, there was an increase from May to November 2020. This rapid recovery of new-onset cases was both due to an interruption of the plateau of cases that usually occur during the summer months and a peak of the occurrence of new cases at the end of the year. This phenomenon has been also described more recently by Kamrath et al. in the DPV registry [22].

For the first quarter of 2021, our data show an increase in the expected number of cases in this period with respect to previous years. We cannot determine whether this increase in the actual number of cases in the first quarter of 2021 is the result of an increase in incidence or whether it is due to a change in monthly distribution as observed in previous months. We can speculate that impact that different COVID waves and associated protective measures may be having in terms of altering other seasonal infections such as flu, respiratory syncytial virus, rhinovirus, or coxsackie, would play a role in these epidemiological abnormalities observed for new diabetes cases. It is still unclear the involvement of COVID in the genesis or progression of type 1 diabetes. A recent report by the. Centers for Disease Control and Prevention (CDC) of the US reported that persons under 18 y with COVID-19 were more likely to receive a new diabetes diagnosis >30 days after the infection than were those without COVID-19 and those with pre-pandemic acute respiratory infections [23].

A limitation of our study is that we cannot provide incidence rates. The representative sample limited to nine tertiary-level hospitals in Spain showed an increase in DKA as a form of presentation, with a higher presentation in children under 4 years of age and changes in the monthly distribution. In addition, our study collects cases only under the age of 14 years old as the majority of pediatric diabetes centers in Spain take care of patients up to this age group. This might be responsible for the differences observed in the findings of other European countries such as Italy or Germany. Further studies are needed to determine whether these epidemiological changes are directly related to SARS-CoV-2, with the modification of hygiene measures, or other factors that are not being considered at the present time.

In conclusion, a change in monthly distribution was noted, as well as an increase in DKA at onset of type 1 diabetes without differences in DKA severity during the first year after the pandemic commencement. Differences in age distribution were observed with an increase in the number of cases in children under 4 years of age. The final causes of these epidemiological changes remain unknown.

## Figures and Tables

**Table 1:** Distribution of new cases by period and by age group.

	Age (Years)		
Period	≤4 Years	5–9 Years	≥10 Years
2015–2019	204 (19%)	424 (39%)	456 (42%)
2020–2021	88 (25%)	108 (30%)	163(45%)
